# Successful Prehospital Extracorporeal Cardiopulmonary Resuscitation: A Comprehensive Case Report and Analysis of the Current Experience and Knowledge

**DOI:** 10.7759/cureus.49975

**Published:** 2023-12-05

**Authors:** Vasileios Leivaditis, Athanasios Papatriantafyllou, Shbiha Khokhar, Bernd Haaf, Inna Kammerer, Michael Kinn, Stefan Hofer, Manfred Dahm

**Affiliations:** 1 Department of Cardiothoracic and Vascular Surgery, Westpfalz-Klinikum, Kaiserslautern, DEU; 2 Department of Anaesthesiology and Critical Care, Westpfalz-Klinikum, Kaiserslautern, DEU

**Keywords:** out-of-hospital cardiac arrest, extracorporeal life support, prehospital ecmo, cardiac arrest, extracorporeal cardiopulmonary resuscitation

## Abstract

This case report describes a prehospital extracorporeal cardiopulmonary resuscitation (ECPR) in a female patient who suffered a sudden cardiac arrest while shopping in a supermarket. The success of this intervention marks the first of its kind in our institution and highlights the potential of prehospital application of extracorporeal membrane oxygenation (ECMO) systems in life-threatening scenarios. Despite the complicated challenges faced in this case, the patient exhibited a favorable neurological outcome. This case underscores the potential of prehospital ECMO in cardiac arrest scenarios and the benefits of a coordinated, multidisciplinary approach. As prehospital ECMO evolves, it offers hope for saving lives in critical situations where rapid intervention is essential.

## Introduction

Cardiac arrest remains a leading cause of mortality worldwide, prompting innovative approaches to enhance survival outcomes. Extracorporeal cardiopulmonary resuscitation (ECPR) has emerged as a potential game-changer in the realm of resuscitative efforts. This intervention involves the rapid initiation of extracorporeal membrane oxygenation (ECMO) support during cardiac arrest, aiming to provide circulatory and respiratory assistance when conventional measures fall short. The foundation of ECPR rests on the principles of venoarterial ECMO, facilitating oxygenation and maintaining systemic perfusion during refractory cardiac arrest scenarios. Over the years, seminal studies have shed light on the theoretical underpinnings and initial clinical applications of ECMO in cardiac arrest, emphasizing its role as a bridge to recovery in select patient cohorts [1]. Concurrently, smaller-scale trials have highlighted the feasibility and potential benefits of prehospital ECMO implementation, marking a pivotal shift in the paradigm of resuscitative strategies [2]. However, the widespread adoption of ECPR and its economic and logistical implications necessitate a comprehensive understanding of patient selection criteria, procedural nuances, and long-term outcomes. The evolving landscape of critical care interventions, underscored by comprehensive reviews, emphasizes the pressing need for evidence-based guidelines and standardized protocols to maximize the efficacy and accessibility of ECPR in diverse healthcare settings [3]. We present a remarkable case of a female patient who suffered a cardiac arrest and was successfully treated with prehospital implantation of an ECMO support system. This case marks a significant milestone as the first of its kind in our hospital.

## Case presentation

**Figure 1:**
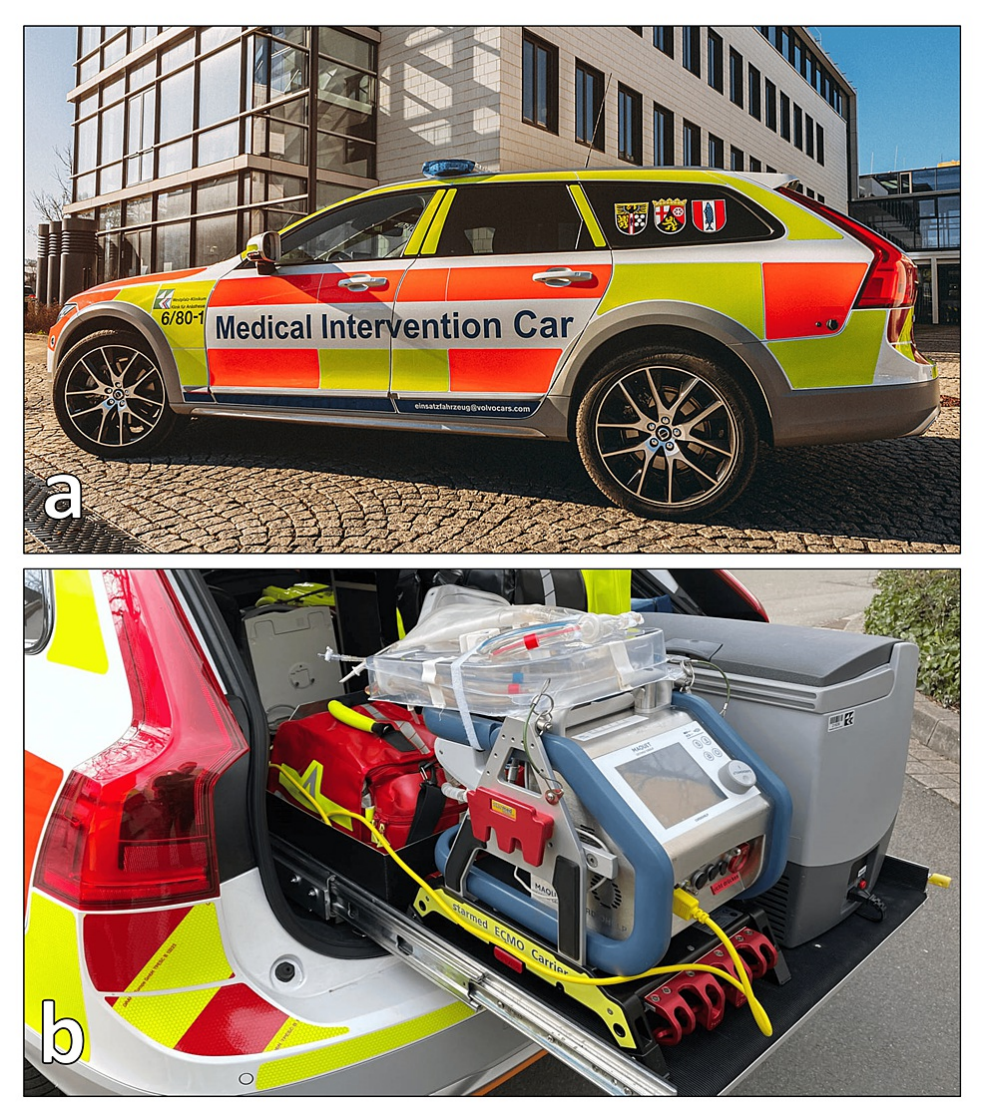
a. The medical intervention car (MIC) utilized within our hospital. b. The extracorporeal life support (ECLS) device which is transported in the MIC.

**Figure 2:**
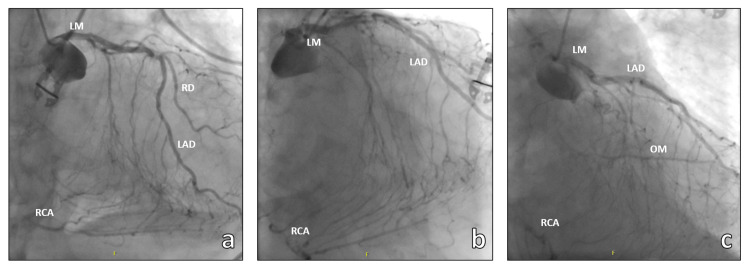
Coronary angiography images showing the severe three-vessel disease with chronic occlusion of RCA and RCX and the collateral network which compensated the myocardial perfusion. LM: Left main, LAD: left anterior descending, RCA: right coronary artery, OM: obtuse marginal, RD: ramus diagonalis

A 66-year-old female patient experienced a collapse while shopping and received prompt external resuscitation. The initially documented cardiac rhythm was ventricular fibrillation. During the early phases of resuscitation and 4 min after the arrival of the emergency team, the medical intervention car (MIC) was summoned. The MIC-Team, comprising an anesthesiologist, a cardiac surgeon, and a clinical perfusionist, was immediately mobilized and arrived at the scene (Figure 1) 18 min after the alarm. A percutaneous arteriovenous ECMO was instituted through the femoral vessels during this intervention. The time from the arrival to cannulation was 21 min. Following stabilization, the patient was subsequently transferred to our hospital. The time interval from the MIC arrival to the transportation to the hospital was 1 h and 19 min. The total out-of-hospital time was 1 h and 42 min. Upon admission, a diagnostic crash computed tomography (CT) scan revealed evidence of coronary disease. Subsequent coronary angiography corroborated the presence of severe three-vessel coronary disease with chronic occlusion observed in both the dominant right coronary artery (RCA) and the small circumflex artery (RCX), precluding any feasible intervention (Figure 2).

Upon transfer to the intensive care unit (ICU), acute ischemia of the left leg was evident post-ECMO application, leading to transfemoral embolectomy and fasciotomy. Due to the chronic occlusion of the superficial femoral artery, implantation of a perfusion sheath for leg perfusion was unfeasible. Central recannulation of the ECMO via the right subclavian artery resolved any perfusion disturbances in the right leg and offered better unloading of the left ventricle. Levosimendan was administered to stabilize cardiac function. Subsequent echocardiography revealed an ejection fraction (EF) of approximately 35%. The patient was further weaned and the ECMO could be removed after seven days.

Ten days postadmission, extubation was attempted; however, due to severe chronic obstructive pulmonary disease, re-intubation followed two days later. Percutaneous dilation tracheostomy was performed. Weaning from mechanical ventilation was initiated, and sedation was discontinued.

The patient displayed no neurological abnormalities after a brief recovery period. She remained alert, responsive, and neurologically intact. Antibiotic therapy with piperacillin/tazobactam was initiated for postoperative pneumonia, escalated to meropenem as inflammatory markers increased, and subsequently discontinued as markers normalized.

As the patient’s condition improved, wound dressings were regularly changed, and a vacuum-assisted closure system was employed. Heart failure management, nutrition, and patient mobilization were implemented. Echocardiography indicated an EF of 35%, obviating the need for an implantable cardioverter-defibrillator as secondary prophylaxis. Following successful weaning, the patient was transferred to a rehabilitation clinic for further therapy.

## Discussion

**Figure 3:**
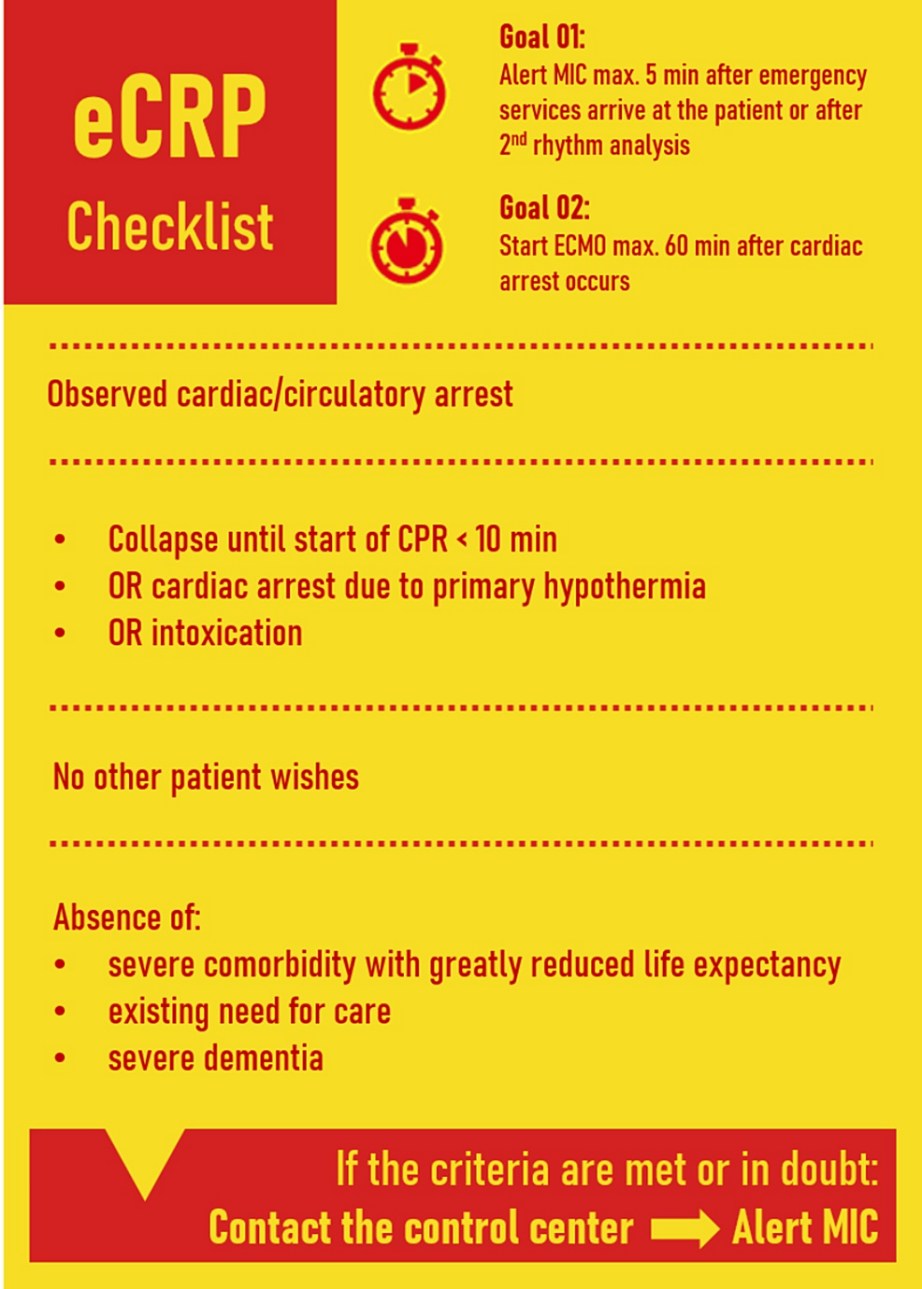
A simplified representation of the protocol utilized in our institution, detailing the appropriate indications and exclusion criteria preceding the application of ECPR. ECPR: Extracorporeal cardiopulmonary resuscitation; MIC: medical intervention car

This case outlines a unique and challenging scenario in which a female patient, experiencing a cardiac arrest, received prehospital implantation of ECMO support. It sheds light on several key aspects of prehospital ECMO use and postresuscitation care as other similar cases described in the current literature [1]. Our hospital deemed prehospital ECMO feasible based on meticulous inclusion and exclusion criteria outlined in Figure 3, defining specific goals and appropriate timeframes for this intervention. These criteria were established to ensure the judicious application of prehospital ECMO, considering factors such as distance from the hospital, expected response times, and patient conditions warranting such advanced intervention. The equipping of the MIC involved a comprehensive process. It included the provisioning of specialized ECMO and surgical equipment, rigorous training of the MIC team members, and the development of robust protocols tailored for prehospital ECMO procedures. Recognizing the time-sensitive nature of successful intervention, our institution adheres to stringent time-to-intervention requirements, emphasizing the importance of swift and coordinated actions for optimal patient outcomes.

The use of ECMO in a prehospital setting is relatively novel and provides a lifeline for patients who experience sudden cardiac arrest outside of a hospital. The financial implications of ECPR differ among health systems and could strain resources if employed without careful consideration. Comprehensive studies ideally should encompass economic assessments, offering valuable insights to healthcare systems regarding the cost-effectiveness of this therapy [2]. This case exemplifies the successful initiation of ECMO during external resuscitation, highlighting the potential to improve outcomes in such dire situations.

Prehospital ECMO, also known as "ECPR", is a rapidly evolving field in critical care medicine. ECMO is traditionally used in the ICU for patients with severe cardiac and respiratory failure, but its role in prehospital settings has gained increasing attention. Current knowledge on prehospital ECMO is derived from a growing body of case reports, small clinical trials, and retrospective analyses [3].

The selection criteria for ECMO candidacy in the prehospital setting are not yet standardized, and criteria often depend on institutional protocols and local expertise [4]. The available evidence does not decisively support or oppose the utilization of ECPR for both out-of-hospital cardiac arrest (OHCA) and in-hospital cardiac arrest (IHCA) in adults and children. The overall quality of evidence from various studies is notably inadequate. This highlights the need for further research to establish clear guidelines and best practices for patient selection [5].

It is important to acknowledge that prehospital ECMO is not without its challenges and potential complications. These include the risk of limb ischemia, bleeding, neurological complications, and infection. The management of these complications is crucial and requires a well-coordinated healthcare team with expertise in ECMO and related critical care interventions [2].

This study case report aims to address the aspect of peripheral limb perfusion following femoral cannulation and the necessity for a distal perfusion cannula (DPC). Previous experience indicates a relatively low risk of lower limb ischemia (LLI) within the initial hours post-implantation [6]. However, there is no observed decrease in the risk of limb ischemia-related events after six hours when comparing bilateral femoral cannulation to unilateral femoral cannulation in peripheral venoarterial ECMO [6]. Despite this, bilateral cannulation is associated with a reduced risk of compartment syndrome/fasciotomy, a lower incidence of bleeding, and vessel repair [6]. Additionally, employing a surgical approach is linked to a low incidence of LLI without an increased risk of in-hospital mortality [7]. Therefore, we propose percutaneous implantation with temporary disregard for limb perfusion at the incident site, followed by DPC implantation upon hospital arrival, and ultimately, the surgical removal of cannulas from the femoral vessels.

Experiences and outcomes

The utilization of prehospital ECMO is associated with varied experiences and outcomes, with success contingent upon several factors, including the patient's clinical condition, the efficacy of the prehospital team, the preparedness of the receiving hospital, and the technical proficiency of ECMO deployment [8]. Published case reports and limited clinical trials suggest that prehospital ECMO may present a viable option for selected patients who have experienced cardiac arrest in the community [3]. Determining suitable candidates for prehospital ECMO involves assessment by trained EMS personnel, considering factors like initial cardiac rhythm and overall health (Figure 3). Protocols guide their decision-making, often in collaboration with receiving hospitals and ECMO specialists.

Controlled, low-risk bias cohort studies indicate a trend toward improved survival with favorable neurological outcomes, but the preponderance of low-quality evidence may contribute an optimistic effect size to ECPR in the context of OHCA survival [9].

The Minnesota Mobile Extracorporeal Cardiopulmonary Resuscitation Consortium, a pioneering initiative targeting out-of-hospital refractory ventricular fibrillation, marked the inception of the first community-wide ECMO-assisted resuscitation program in the United States. This program achieved a 100% success rate in cannulation, with functionally favorable survival rates of 43% observed at both hospital discharge and three months, underscoring its safety and efficacy [10].

Significantly, positive outcomes are frequently associated with prompt treatment initiation, with studies indicating that shorter "low-flow" times (the duration between cardiac arrest and ECMO initiation) are linked to better results [11]. Efficient coordination between prehospital and hospital teams is crucial for optimal outcomes.

The Prague Out-of-Hospital Cardiac Arrest (OHCA) Study revealed that for individuals experiencing refractory OHCA, the combination of early intra-arrest transport, ECPR, and invasive assessment and treatment did not result in a substantial improvement in survival with a neurologically favorable outcome at the 180-day mark when compared to standard resuscitation practices [12]. It is noteworthy that the trial might have been inadequately powered to identify a clinically significant difference. However, the secondary analysis of the randomized refractory OHCA trial indicated that ECPR was associated with enhanced 180-day survival among patients who did not experience prehospital return of spontaneous circulation [13].

Finally, in the Inception Clinical Trial involving 160 individuals with refractory out-of-hospital cardiac arrest, a comparison between ECPR and conventional CPR revealed comparable effects on survival with a favorable neurological outcome [14].

Future directions and perspectives

As the field continues its evolution, there is an evident imperative for additional research, the establishment of standardized protocols, and the dissemination of best practices. The undertaking of prospective clinical trials and large-scale studies is crucial to elucidate the specific patient populations that are most likely to derive significant benefits [3].

This case exemplifies the success achieved through a multidisciplinary approach to patient care, involving teams from cardiovascular, surgical, anesthesiology, and critical care disciplines. Close coordination and expertise across diverse medical domains proved instrumental in effectively addressing the numerous challenges encountered [15].

The favorable neurological outcome observed in this case underscores the efficacy of the selected therapeutic interventions. The combination of prompt resuscitation, ECMO support, and postresuscitation care likely contributed to this positive outcome [8]. This particular case adds valuable evidence to the expanding body of literature supporting the role of prehospital ECMO and advocates for further exploration in this promising field.

As we look forward, future research endeavors in this domain should prioritize refining the criteria for patient selection, optimizing the timing of interventions, and assessing long-term outcomes. Standardized protocols and guidelines should be established through collaborative efforts, ensuring consistency and reproducibility across diverse healthcare settings. Additionally, the integration of innovative technologies and continuous training programs will be pivotal in enhancing the proficiency of prehospital ECMO implementation. The ongoing collaboration between multidisciplinary teams, coupled with a commitment to evidence-based practices, will contribute to the continual advancement of prehospital ECMO as a life-saving intervention in critical care scenarios.

## Conclusions

This report serves as a pioneering example of prehospital ECMO support. The successful outcome underscores the importance of rapid and well-coordinated medical responses, even in the face of significant challenges. With ongoing research and the accumulation of more clinical experiences, the application of prehospital ECMO is likely to become more refined and accessible, potentially saving more lives in situations where time is of the essence.
